# The positive impact of informal spousal caregiving on the physical activity of older adults

**DOI:** 10.3389/fpubh.2022.977846

**Published:** 2022-12-16

**Authors:** Hua Zan, Su Hyun Shin

**Affiliations:** ^1^Center on the Family, University of Hawai'i at Mānoa, Honolulu, HI, United States; ^2^Department of Family and Consumer Studies, University of Utah, Salt Lake City, UT, United States

**Keywords:** spousal caregiving, instrumental variable (IV), Health and Retirement Study (HRS), United States, subgroup differences

## Abstract

**Introduction:**

Although physical activity (PA) is crucial for health, the literature is mixed about how individuals' PA decisions are affected by their spouses. To fill this gap, we examined the extent to which providing care for one spouse affects the PA of the other spouse among those aged 50 or older in the United States.

**Methods:**

We analyzed 9,173 older adults living with their spouses or partners from the 2004 to 2016 waves of the Health and Retirement Study. To identify the causal effect of spousal caregiving on the PA of older adults, we estimated individual-fixed effects models using a two-stage least squared instrumental variable approach with spousal falls as our instrument. We also estimated the models by splitting the sample by gender and race/ethnicity to identify heterogeneous impacts of spousal caregiving on PA decisions among subgroups.

**Results:**

We found that a one percentage point increase in the probability of providing care to spouses led to an increase in the probability of initiating moderate or vigorous PA (MVPA) by 0.34–0.52 percentage points. This effect was salient, especially among female and non-Hispanic white older adults.

**Discussion:**

Caregiving experience might provide opportunities to learn about caregiving burdens and trigger an emotional response about the salience of an event (i.e., they need care in the future). Older caregivers might start MVPA in an effort to improve or maintain their health and avoid burdening their families for caregiving in the future. This study demonstrated spousal influence on PA. Instead of delivering PA-promotion information (e.g., the harm of sedentary lifestyle and benefits of regular PA) to individuals, risk communication and education efforts on PA promotion might be more effective considering the family context. Family events such as health shocks or the emergence of caregiving needs from family members provide windows of opportunities for intervening. Subgroup differences should also be considered in targeted interventions.

## 1. Introduction

Physical activity (PA) is associated with a variety of health benefits and has a critical role in the etiology and prevention of many chronic diseases, such as cancer, coronary heart disease, and obesity (1). Because physical inactivity is the fourth leading risk factor for death in the world (2), there is growing interest in people's PA decisions. There are also substantial economic costs (3)—$53.8 billion worldwide in 2013 (4)—for being inactive over long periods.

Families are an important factor for promoting healthy activities (5, 6), often shaping PA patterns by providing constraints and/or support (7). For example, family responsibilities such as caregiving not only directly limit the time for PA but can also change it indirectly by affecting caregivers' weight and health (8). By encouraging and monitoring the PA of those they care for, families often provide social support for and control over their PA patterns (9).

This study focused on the role that informal caregiving plays in PA among older adults. The rationale is that caregiving activities are largely shouldered by families (10) due to the cost of market-provided formal care and the limited public assistance programs in the U.S. (11). In 2015, 34.2 million or 14.3% of Americans were informal caregivers of adults aged 50 or older, and the prevalence of caregiving rose to 16.8% in 2020 (12). The growing demand for informal care raises questions about its impact on the caregiver's health and wellbeing (13). While the physical and emotional toll on caregivers has been well-documented in the literature (14–17), caregiving is not always a negative experience, especially when the burden is light (18). However, in 2020, 40 and 16% of caregivers provided high- and medium-burden care, respectively (12). Given the heavy burdens on many family caregivers, it is critical to assess the impact of caregiving on caregivers' PA decisions.

**Figure 1:**
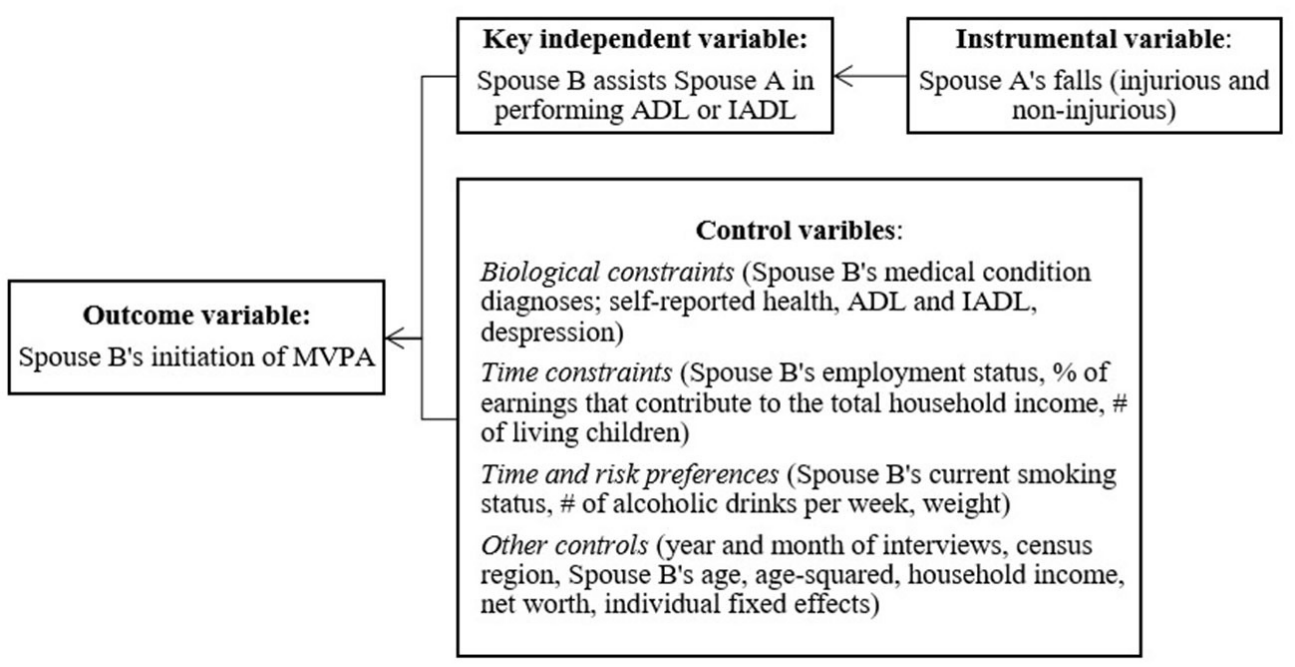
A conceptual framework and variables. MVPA, moderate-to-vigorous physical activity; ADL, activities of daily living; IADL, instrumental activities of daily living.

Caregiving situation can vary by the type of relationship between caregivers and care recipients; and caregiving experiences and caregiver needs can be different for spouse caregivers vs. other caregivers such as adult children (19). Often living with care recipients, spouse caregivers provide more hours of care as the primary caregiver (20, 21) and experience more financial and health issues compared to adult child caregivers (22, 23). Considering such differences, this study focused on spousal caregiving. To understand the PA decisions of one spouse (Spouse B) subject to the influence of caregiving to the other spouse (Spouse A), we used the sleeping, leisure, occupation, transportation and home production (SLOTH) model of time allocation proposed by Cawley (8) as the theoretical framework. Figure 1 illustrates the conceptual framework for this study. Caregiving to one spouse (Spouse A) may influence the other spouse (Spouse B)'s PA through affecting one or a combination of: *time allocation, energy level, health*, and *preferences*. Spousal caregiving decreases PA through its impact on *time allocation* and *energy level*. Caregiving decreases the caregiver's time spent on paid work and leisure activities (24), including PA (25, 26), especially among co-residing (27) and women caregivers (24). Some caregivers also curtail PA because they are too tired from providing care (28–30).

It is, however, unclear how spousal caregiving would affect PA through *health*. The literature indicates that caregiving activities are often associated with negative health outcomes for caregivers (14, 15, 24). A health decline may motivate caregivers to engage in more PA to improve their own health (31, 32), as well as to cope with the stress induced by providing care (33). Meanwhile, some people are less likely to be physically active when they are stressed (34, 35), and mental health problems are often associated with risky health behaviors (36), including adopting sedentary lifestyles.

PA decisions are also affected by *preference*. It is well-documented that someone who is future-oriented tends to spend more time on PA (37, 38). Also, previous studies identified that individuals' own past experiences and the observations of others' experiences play a role in their decision making such as taking precautionary measures (39, 40). As such, caregiving experience can change caregivers' preference on their own future care options (e.g., whether they prefer receiving informal care from their family or purchasing long-term care insurance to pay for costs associated with long-term care). One study found past caregiving experience increases the future intention to purchase long-term insurance (11). In fact, knowing someone who suffered from illness had a stronger effect on the intentions to purchase long-term insurance than one's past experience did, which suggests that anticipatory emotions triggered by the vividness of others' experience (vicarious experience) motivated the intentions to purchase (39). As such, vicarious experience through spousal caregiving can motivate a change in caregivers' PA in an effort to improve or maintain their own health and delay the onset of medical conditions requiring long-term care.

Neither the SLOTH model nor the literature concerning the impact of informal caregiving on PA is conclusive (41). While some studies found higher PA among caregivers (42, 43), others found lower PA (44–46), and still others found no difference between caregivers and non-caregivers (47–49). Some plausible explanations for this include differences in PA measures (e.g., leisure-time PA vs. total PA and self-reported vs. accelerometer-measured PA), caregiving measures (e.g., caregiving provisions, caregiving intensity, and caregiving frequency), sample sizes, sample design (e.g., cross-sectional vs. longitudinal), and methodologies (e.g., descriptive vs. multivariate analysis).

Previous studies largely focused on the correlation between informal care and PA and relied on cross-sectional data. They did not establish causality. Unobserved characteristics can make people more or less likely to be caregivers and physically active. For example, those with flexible working hours are more likely to engage in PA (50) and be caregivers as needs arise. To identify a causal relationship, this study used longitudinal data, an instrumental variable approach, and individual-fixed effects models to control for unobserved time-invariant individual characteristics such as race/ethnicity and genetics. We used the 2004–2016 waves of the Health and Retirement Study (HRS) to examine the effect of providing care for one spouse on the initiation of moderate or vigorous PA (MVPA) of the other spouse among adults aged 50 or older. Understanding determinants of a change in PA is crucial for locating opportunities for interventions to promote PA (51). We focused on MVPA for its demonstrated health benefits to older adults (52) and a greater effect on a mortality risk reduction compared to light PA (53). Starting MVPA is an important change in health behavior because research has shown even a low level of MVPA had a moderate effect on a mortality risk reduction among older adults (52). Additionally, given the different PA patterns and informal caregiving by sex (54) and race/ethnicity (55), we also examined the extent to which the effect of spousal caregiving on PA differs by these characteristics of older adults.

## 2. Materials and methods

### 2.1. Data and sample

We used data from the 2004–2016 waves of the Health and Retirement Study (HRS). The HRS has surveyed biennially a representative sample of approximately 20,000 Americans aged 50 or older and their spouses since 1992 (56). The survey provides information on physical, mental, and cognitive health, as well as demographic and socioeconomic characteristics of Americans and their families. We included data starting from 2004 because HRS has collected PA information of the respondents and their spouses consistently since that year.

Our sample included 9,173 unique individuals (26,227 wave-individuals) aged 50 or older who reside with their spouses or partners. Table 1 summarizes their characteristics. The average age was 71, more than half were female (59%), a majority was non-Hispanic white (82%), had a high school degree or higher (86%), were either retired or not working (86%), had some health insurance (94%), and owned a home (92%). While most reported good or better health (79%) and were non-smokers (93%), 7 out of 10 were overweight or obese.

**Table 1:** Sample characteristics.

**Variables**	**Mean**	**S.D**.	**Variables**	**Mean**	**S.D**.
**Dependent variable**			Arthritis	0.679	0.449
PA initiation	0.052	0.158	Psychiatric problem	0.134	0.346
**Explanatory variable**			Memory-related disease	0.017	0.151
Informal care for spouse	0.114	0.287	CES-D score	1.022	1.543
Help performing ADLs	0.068	0.252	Difficulties with ADLs		
Help performing IADLs	0.093	0.291	Walking across a room	0.040	0.192
**Instrument**			Dressing	0.068	0.237
Fall	0.321	0.383	Bathing/taking a shower	0.034	0.185
Non-injurious fall	0.229	0.337	Eating	0.016	0.122
Injurious fall	0.092	0.234	Getting in/out of bed	0.033	0.173
**Control variables**			Using the toilet	0.036	0.163
Female	0.587	0.496	Difficulties with IADLs		
Race/ethnicity			Using a map	0.110	0.296
White	0.819	0.409	Using the phone	0.024	0.146
Black	0.092	0.305	Managing money	0.030	0.172
Hispanic	0.053	0.246	Taking medications	0.016	0.125
Other	0.019	0.143	Shopping for groceries	0.056	0.228
Educational attainment			Preparing hot meals	0.038	0.191
Less than high school	0.142	0.377	Employment status		
High school	0.376	0.481	Employed	0.138	0.323
Some college	0.227	0.419	Retired	0.786	0.378
Bachelor's degree	0.254	0.426	Not working	0.077	0.244
Age	71.749	7.480	% contribution to income	0.072	0.167
Self-reported health			Health insurance ownership	0.941	0.221
Poor	0.048	0.212	Home ownership	0.921	0.285
Fair	0.165	0.335	Household income^a^	81,250	103,103
Good	0.347	0.386	Household net worth^a^	786,210	1,399,038
Very good	0.343	0.384	Number of children	3.469	2.147
Excellent	0.097	0.244	Current smoker	0.072	0.270
Medical conditions			Number of drinks per week	2.365	4.956
High blood pressure	0.636	0.462	Weight status		
Diabetes	0.223	0.410	Under-weight	0.011	0.101
Cancer	0.189	0.373	Normal	0.287	0.423
Lung disease	0.096	0.293	Over-weight	0.394	0.436
Heart problem	0.284	0.433	Obese	0.308	0.434
Stroke	0.062	0.243			
Obs.	26,227				
*N*	9,173				

Unweighted. 2004–2016 HRS.

^a^2016 dollars.

### 2.2. Variables

Figure 1 provides a conceptual framework for all the variables used in this study. Following the literature (57), we used the initiation of moderate or vigorous PA (MVPA) as an outcome variable. The HRS asks respondents about the frequency (“every day,” “more than once a week,” “once a week,” “one to three times a month,” “hardly ever or never”) and intensity (vigorous, moderate, and mild) of PA. Examples provided to the respondents for vigorous activities include running or jogging, swimming, cycling, aerobics or gym workout, tennis, or digging with a spade or shovel. Moderate activities include gardening, cleaning the car, walking at a moderate pace, dancing, floor or stretching exercises. We excluded mild activities because the HRS' definition of such activities includes primarily house chores, such as vacuuming, laundry, and home repairs. To create an indicator for the MVPA initiation, we coded it as one if an older adult reported engaging in MVPA at least one to three times a month at the current period (*t*), but not in the previous period (*t – 1*), and zero if otherwise. In our sample, approximately 5% started MVPA during the study period (Table 1).

The main explanatory variable was whether a respondent provided informal care for their spouse in the current period (*t*). Following the literature (30, 58), we coded it as one if a respondent assisted their spouse in performing activities of daily living (ADL) or instrumental activities of daily living (IADL) or identified as a helper in the survey and zero if otherwise. In our sample, 11% were identified as informal caregivers for their spouses (Table 1). As a robustness test to explore any heterogeneity in the relationship by types of assistance (ADL or IADL), we also included assisting in performing ADLs and IADLs, respectively as indicators of spousal informal caregiving (see Supplementary Table 2). Approximately 7% and 9% helped their spouses' ADLs and IADLs (Table 1).

To predict the endogenous variable of spousal caregiving, we used spousal falls as our instrument, coding it as one if a respondent's spouse reported having fallen in the previous two years, and zero if otherwise. A valid instrument must meet three assumptions (59, 60): it is related to spousal caregiving (relevance assumption); it does not share common causes with the MVPA initiation (independence assumption); and it does not have a direct effect on the MVPA initiation, only indirectly through spousal caregiving (exclusion restriction). Spousal falls met the relevance assumption because the literature suggests that falls are associated with ADL/IADL difficulty (61, 62), morbidity and mortality (63, 64), and family members are often responsible for caregiving without preparation (65).

We further categorized spousal falls into injurious and non-injurious falls based on whether there was a need for medical treatment. We did so because severe falls might be associated with admission to long-term care facilities (66), which might reduce the need for informal care. We thus expected non-injurious falls to be more likely associated with informal caregiving than injurious falls. In our sample, 22% and 9% of spouses experienced non-injurious and injurious falls, respectively (Table 1).

Following the SLOTH model by Cawley (8), we included a rich set of covariates to capture the individual's biological and time constraints, and time and risk preferences (see Supplementary Table 1 for detailed variable description). For biological constraints, we controlled for a physician's diagnosis of nine medical conditions (e.g., high blood pressure), self-reported health status, difficulties in performing ADL and IADL, and Center for Epidemiological Studies Depression (CES-D) scores for mental health. For time constraints, we included the respondents' employment status, the percentage of their earnings that contributed to total household income, and the number of living children. For time and risk preferences, we controlled for health behaviors, including current smoking status, the number of alcoholic drinks per week, and weight status. Other controls included the year and month of interviews, census region, and the basic demographic and socioeconomic status of respondents and households (e.g., age and age-squared, household income, and net worth). We also included individual-fixed effects to control for time-invariant unobservables (e.g., genetics).

### 2.3. Empirical model

To examine the effects of providing care for spouses (*IC*_*it*_) on older adults' MVPA initiation (*MVPA*_*it*_), we first estimated individual-fixed effects linear regression models, as follows:


(1) $$\begin{array}{l} {MVPA}_{it}\;=\;\beta_{1}{IC}_{it}\;+\;\beta_{2}X_{it}\;+\;i_{i}\;+\;y_{y}\;+\;m_{m}\;+\;r_{r}\;+\;\varepsilon_{it} \end{array}$$

where *MVPA*_*it*_ denotes a respondent *i*'s MVPA initiation in year *t*. *IC*_*it*_ is an indicator of whether the respondent *i* provides spousal care in year *t*. *X*_*it*_ denotes a vector of individual and household characteristics. *i*_*i*_, *y*_*y*_, *m*_*m*_, and *r*_*r*_ are individual-, year-, month-, and region-fixed effects, respectively. Although the dependent variable is a binary indicator, we used a linear probability model, which enabled us to compare the OLS estimates from Equation (1) with instrumental variable (IV) two-stage least squares (2SLS) estimates from the following equations:


(2) $$\begin{array}{l} 1\text{st stage}:\;{IC}_{it}\;=\;\alpha_{1}Z_{it}\;+\;\alpha_{2}X_{it}\;+\;i_{i}\;+\;y_{y}\;+\;m_{m}\;+\;r_{r}\;+\;\epsilon_{it} \end{array}$$

(3) $$\begin{array}{l} 2\text{nd stage}:\;{MVPA}_{it}\;=\;\gamma_{1}I{\hat{C}}_{it}\;+\;\gamma_{2}X_{it}\;+\;i_{i}\;+\;y_{y}\;+\;m_{m}\;+\;r_{r}\;+\;\delta_{it} \end{array}$$

In the first stage, we predicted a respondent *i*'s probability of providing spousal care in year *t* by an instrument *Z*_*it*_—spousal falls—after controlling for covariates *X*_*it*_ and other fixed effects. In the second stage, the predicted probability of providing spousal care ($I{\hat{C}}_{it}$) obtained from Equation (2) estimated the respondent *i*'s probability to start MVPA in year *t*. Because couples tend to have similar health behaviors (32), including both the respondents and their spouses in the model may overestimate the impact of informal caregiving on the MVPA initiation. Therefore, we re-estimated Equations (2) and (3) by restricting our sample to family respondents as a robustness test.

Because the decisions of older adults about informal caregiving and PA may differ by the severity of spousal falls, we also re-estimated Equations (2) and (3) using non-injurious and injurious falls separately as the instrument *Z*_*it*_. We also partitioned the full sample by sex (male vs. female) and race/ethnicity (non-Hispanic white vs. non-white) and re-estimated Equations (2) and (3) to explore subgroup differences.

To check the validity of our instrument, we tested the extent to which spousal falls meet the relevance assumption, independence assumption, and exclusion restriction. For the relevance condition, the first stage result from Equation (2) showed whether spousal falls were associated with spousal caregiving. For the independence assumption, we investigated whether spousal fall variables were independent of factors that can be correlated with PA by regressing spousal falls on the same set of covariates and using individual-fixed effects regression estimators. If spousal falls are unrelated to the covariates, it is reasonable to assume that spousal falls are unexpected and present a shock to individuals. For the exclusion restriction, we tested two alternative pathways that spousal falls may have a direct effect on the MVPA initiation (i.e., not through spousal caregiving). One pathway is that a respondent might start MVPA because their spouse did, following a physician's recommendation, and couples tend to engage in PA together (co-PA). To test this pathway, we regressed on spousal falls on the spousal initiation of MVPA. The other pathway for how spousal falls might influence PA was that individuals might change their evaluation of their health from spousal falls (not through caregiving experiences) and change their PA accordingly. To test whether this pathway exists, we used self-assessed longevity and nursing home care use because the evidence shows that people updated their subjective life expectancies and nursing home entries after exposure to health shocks (67, 68). Specifically, we used the respondents' subjective probability of living another 10 years and entering a nursing home in the next 5 years as the dependent variable in the first stage and the predicted values of the probabilities as the independent variable in the second stage.

## 3. Results

### 3.1. Main results

Table 2 presents the results from the model using Equation (1) with the full sample (Specification I) and IV 2SLS models using Equations (2) and (3) with the full sample (Specification II) and family respondents only (Specification III). The results from the OLS model show that whether an older adult provided care for his/her spouse was not related to the older adult's MVPA initiation. The IV 2SLS models, however, produced interesting results. Specifically, in the first stage with the full sample, we found that spousal falls (both injurious and non-injurious falls) increased the probability of respondents' caregiving by 3.03 percentage points. In the second stage, a one percentage point increase in the probability of providing spousal care led to an increase in the probability of individuals starting MVPA by 0.34 percentage points. When we restricted our sample to family respondents only, the effect of spousal care on MVPA initiation was even larger. Because the analysis based on the full sample produces more statistically significant conservative results (i.e., the coefficient for informal caregiving is significant at *p* < 0.05 with the full sample vs. at *p* < 0.01 with family respondents only), we used the full sample in further analyses.

**Table 2:** Effects of providing informal care for spouse on MVPA initiation.

	**(I) OLS**	**(II) IV**		**(III) IV**	
	**Full sample**	**Full sample**		**Family respondents**	
		**1st**	**2nd**	**1st**	**2nd**
	**Coef**.	**Coef**.	**Coef**.	**Coef**.	**Coef**.
	**(S.E.)**	**(S.E.)**	**(S.E.)**	**(S.E.)**	**(S.E.)**
Provide care for spouse	0.0064				
	(0.0072)				
Spousal fall		0.0303^***^		0.0377^***^	
		(0.0046)		(0.0065)	
Predicted probability of informal care for spouse			0.3379^*^		0.5174^**^
			(0.1510)		(0.1854)
Obs.	26,227	26,227	26,227	14,844	14,844
*N*	9,173	9,173	9,173	5,094	5,094
R^2^	0.0177				
Cragg-Donald Wald *F* statistic		43.51		34.01	

Specification (I) = individual-fixed effects regression estimators. Specifications (II) and (III) = individual-fixed effects 2SLS IV estimators. Covariates are controlled in all specifications.

^*^p < 0.05, ^**^p < 0.01, ^***^p < 0.001.

Supplementary Table 2 presents the results of the robustness test on whether the positive effect of spousal care driven by spousal falls on the probability of initiating MVPA is heterogeneous by types of spousal care (i.e., assist in performing ADLs vs. IADLs). The results show that the positive effect is robust across care types. A one percentage point increase in the probability of helping spouses perform ADLs (Specification I) and IADLs (Specification II) led to increases in the likelihood of starting MVPA by 0.30 and 0.47 percentage points, respectively.

Table 3 presents the results from IV 2SLS models using non-injurious falls (Specification I) and injurious falls (Specification II) as the instrument, respectively. In the first stage, a spouse's non-injurious and injurious falls led to an increase in the probability of providing care to the other spouse by 2.17 and 2.54 percentage points, respectively. In the second stage, a one percentage point increase in the probability of spousal caregiving led to an increase in the probability of a respondent starting MVPA by 0.62 percentage points using non-injurious falls as the instrument, but no effect was found when we used spousal injurious falls as the instrument. Consistent with our expectations, the results suggest that the causal effect of providing spousal care on the MVPA initiation is likely driven by non-injurious spousal falls rather than injurious falls. Therefore, the following analyses presented in Section 3.3 used non-injurious falls as the instrument.

**Table 3:** Effects of providing informal care for spouse on MVPA initiation by spousal fall types.

	**(I) Non-injurious fall**		**(II) Injurious fall**	
	**1st**	**2nd**	**1st**	**2nd**
	**Coef**.	**Coef**.	**Coef**.	**Coef**.
	**(S.E.)**	**(S.E.)**	**(S.E.)**	**(S.E.)**
Spousal fall	0.0217^***^		0.0254^***^	
	(0.0049)		(0.0069)	
Informal care for spouse		0.6224^*^		−0.1389
		(0.2537)		(0.2573)
Obs.	26,227	26,227	26,227	26,227
*N*	9,173	9,173	9,173	9,173
Cragg-Donald Wald F statistic	19.55		13.61	

Individual-fixed effects 2SLS estimators. Covariates are controlled in all specifications.

^*^p < 0.05, ^**^p < 0.01, ^***^ p < 0.001.

### 3.2. Validity of the instrument

Our results indicated that the instrument satisfied the relevance assumption, independence assumption, and exclusive restriction. The first stage result showed that spousal falls were positively associated with caregiving (see Tables 2, 3), which indicates that the instrument meets the first condition. The Cragg-Donald Wald F statistic was larger than the general critical value of 10 and the Stock-Yogo's critical value of 16.38 (at 10% maximal IV size), indicating that our instrument was not weakly identified (69, 70). It was also exactly identified because we used one instrument (i.e., spousal fall) for one endogenous regressor (i.e., informal caregiving).

Our results also showed that non-injurious spousal falls were unlikely to relate to most characteristics of respondents, whereas any spousal falls and injurious spousal falls were associated with more characteristics, such as age (see Supplementary Table 3). Non-injurious falls are not meaningfully correlated with other factors that might be related to the MVPA initiation of older adults.

In addition, we tested two alternative pathways through which spousal falls might affect the initiation of MVPA (not through spousal caregiving): (1) co-PA and (2) changing evaluation of one's health from spousal falls. Our results suggest neither pathway explained a respondent's initiation of MVPA. Specifically, the results in Table 4 show that spouses who experienced falls did not start MVPA themselves. The results in Table 5 show that individuals did not change their evaluation of health (i.e., update their subjective probability of living anther 10 years or entering a nursing home in the next 5 years) from spousal falls and then change their PA accordingly. The above tests have provided evidence that our instrument met the exclusion restriction.

**Table 4:** Spousal fall and spousal initiation of MVPA.

	**Fall**		**Non-injurious fall**		**Injurious fall**	
	**(I)**	**(II)**	**(III)**	**(IV)**	**(V)**	**(VI)**
**DV** = **Spousal initiation of PA**	**Coef**.	**Coef**.	**Coef**.	**Coef**.	**Coef**.	**Coef**.
	**(S.E.)**	**(S.E.)**	**(S.E.)**	**(S.E.)**	**(S.E.)**	**(S.E.)**
Spousal fall	0.0037	0.0022	0.0037	0.0001	0.0012	0.0054
	(0.0043)	(0.0033)	(0.0046)	(0.0036)	(0.0065)	(0.0053)
Controls	No	Yes	No	Yes	No	Yes
Obs.	26,227	26,227	26,227	26,227	26,227	26,227
N	9,173	9,173	9,173	9,173	9,173	9,173
R^2^	0.0000	0.0097	0.0000	0.0098	0.0000	0.0097

Fixed-effect regression estimators. Covariates are controlled in all specifications.

**Table 5:** Tests for a direct learning effect from spousal falls.

	**(I) Fall**		**(II) Non-injurious fall**		**(III) Injurious fall**	
	**1st**	**2nd**	**1st**	**2nd**	**1st**	**2nd**
	**Coef**.	**Coef**.	**Coef**.	**Coef**.	**Coef**.	**Coef**.
	**(S.E.)**	**(S.E.)**	**(S.E.)**	**(S.E.)**	**(S.E.)**	**(S.E.)**
**Panel A: Learning effects through life expectancy**						
Spousal fall	0.0041		0.0032		0.0030	
	(0.0123)		(0.0131)		(0.0183)	
Probability of living another 10 years		2.4717		4.2617		−1.1881
		(7.3670)		(17.5644)		(7.4987)
Obs.	22,418		22,418		22,418	
*N*	6,219		6,219		6,219	
Cragg-Donald Wald *F* statistic	0.114		0.059		0.028	
**Panel B: Learning effects through subjective probability of moving to a nursing home in 5 years**						
Spousal fall	−0.0209		−0.0355*		0.0222	
	(0.0165)		(0.0176)		(0.0244)	
Probability of moving a nursing home		−0.5977		−0.3991		0.0146
		(0.5207)		(0.2443)		(0.3211)
Obs.	18,655		18,655		18,655	
*N*	5,358		5,358		5,358	
Cragg-Donald Wald *F* statistic	1.629		4.090		0.829	

Fixed-effect two-stage least squares (2SLS) instrumental variable (IV) estimators. The dependent variables of the second-stage are the respondent's MVPA initiation. The dependent variables of the first-stage in Panel A and B are the probabilities of living another 10 years and moving to a nursing home in the next 5 years, respectively. Covariates are controlled in all specifications. ^*^p < 0.05, ^**^p < 0.01, ^***^p < 0.001.

### 3.3. Heterogeneous effects

Table 6 presents individual-fixed effects 2SLS IV estimators after splitting the sample by sex (Specification I) and race/ethnicity (Specification II). We found a causal relationship between a respondent's informal caregiving and their initiation of MVPA primarily for females and non-Hispanic whites. A one percentage point increase in the probability of providing spousal care led to the increase in the probability of starting MVPA for females and non-Hispanic whites by 0.77 and 0.66 percentage points, respectively. However, we did not identify effects among male and non-white older adults.

**Table 6:** Effects of providing informal care for spouse on MVPA initiation by respondent's sex and race/ethnicity.

	**(I) Male**		**(II) Female**	
	**1st**	**2nd**	**1st**	**2nd**
	**Coef**.	**Coef**.	**Coef**.	**Coef**.
	**(S.E.)**	**(S.E.)**	**(S.E.)**	**(S.E.)**
**Panel A: By sex**				
Spousal non-injurious fall	0.0202^**^		0.0216^**^	
	(0.0074)		(0.0065)	
Informal care for spouse		0.4188		0.7691^*^
		(0.3528)		(0.3751)
Obs.	9,563	9,563	16,664	16,664
*N*	2,761	2,761	6,412	6,412
Cragg-Donald Wald *F* statistic	7.36		10.87	
	**(I) Non-Hispanic white**		**(II) Non-White**	
	**1st**	**2nd**	**1st**	**2nd**
	**Coef**.	**Coef**.	**Coef**.	**Coef**.
	**(S.E.)**	**(S.E.)**	**(S.E.)**	**(S.E.)**
**Panel B: By race/ethnicity**				
Spousal non-injurious fall	0.0227^***^		0.0206	
	(0.0053)		(0.0144)	
Informal care for spouse		0.6553^*^		0.1890
		(0.2591)		(0.7198)
Obs.	19,492	19,492	6,735	6,735
*N*	5,255	5,255	3,918	3,918
Cragg-Donald Wald *F* statistic	18.7		2.05	

Individual-fixed effects 2SLS IV estimators. Covariates are controlled in all specifications. ^*^p < 0.05, ^**^p < 0.01, ^***^p < 0.001.

## 4. Discussion

Using a longitudinal dataset of the 2004–2016 HRS, we found that providing care to one spouse led to an increase in the probability of starting MVPA for the other spouse. This study should not be directly compared to previous studies identifying negative (44–46), positive (42, 43), or no associations between informal caregiving and PA (47–49) because our study is different from the literature in several ways. We estimated a plausibly causal relationship using longitudinal data, and our outcome is MVPA initiation among older adults. The data do not allow us to test the underlying mechanism for the positive effect of spousal caregiving on PA due to a lack of relevant information in the dataset. However, caregiving experience possibly informs caregivers of caregiving burden (e.g., high financial costs and physical/mental health consequences) and triggers an emotional response (i.e., vicarious experience). They may realize that what happened to their spouses could happen to themselves. Older adults may prefer not burdening the family with caregiving duties in the future, and therefore, change their behavior (i.e., start MVPA) in an effort to improve or maintain their health. This explanation is consistent with the literature showing that concerns about burdening families influence the preferences of older adults, especially those with chronic illnesses (71), on their future long-term care options (formal care vs. informal care) and that past and current care experiences play an important role in forming those preferences (72).

For subgroup differences, we only found positive impacts of spousal caregiving on the MVPA initiation among female and non-Hispanic white older adults. This result suggests that women are more responsive than men to caregiving experience and adjust their PA accordingly. Since women tend to be the primary caregiver, caregiving experience may inform women of caregiving burden and trigger emotional responses about the risk of needing informal care in the future. Women may also respond by increasing PA to avoid burdening family members. When care needs arise, however, men may expect to receive care from family members (72, 73), especially from wives or daughters, due to gender norms and societal expectations. Thus, men might be less likely than women to consider care options other than informal care even after providing care to their spouses. Therefore, they might be less motivated to start MVPA to prevent or delay health problems that might require care from family members.

The differential effects by race/ethnicity may reflect cultural differences in who is expected to be a caregiver. In a collectivist culture that emphasizes the family over the individual (74), non-white families may expect children to provide care for their older parents. This is supported by the data showing that a higher percentage of non-white caregivers are adult children compared to white caregivers (75, 76). Additionally, spouses in non-white families can often access support from non-family members, such as relatives, friends, and neighbors (77, 78). Thus, given the cultural expectation and social norms on informal care, non-white older adults may be less motivated compared to whites to start MVPA in an effort to prevent or delay health problems that might burden family members for care provision.

This study has a few limitations. First, although the instrument meets the relevance assumption and exclusion restriction statistically, our IV approach might not be perfect. There could be unobserved confounding factors related to the pathway through which Spouse B's caregiving for Spouse A because of Spouse A's fall increases Spouse B's probability of starting MVPA. Although this study attempted to control for all the potential observables correlated with this pathway (e.g., individual-fixed effects), time-variant unobservables might explain such relationship. Second, our results might be driven by an increase in caregiving-related PA if some survey respondents considered caregiving activities as PA (42). However, because we excluded mild PA in our analysis, we believe that this possibility is reduced, though not eliminated. Third, it should be noted that an IV approach does not ensure the external validity of our findings. Because we used variations in spousal caregiving explained by spousal falls only, the positive impact of spousal caregiving on the MVPA initiation of older adults may not be extended to spousal caregiving driven by other factors, such as hospitalization (79). Fourth, while this study focused on whether spousal caregiving affects MVPA initiation, future research can extend it by examining other types of relationship such as caregiving from adult children or friends, or by determining the impact informal caregiving on other PA measures, such as a duration of PA, PA changes, and leisure-time PA. For example, to improve PA intervention efficacy, one direction for future research is to identify factors associated with the maintenance of positive PA changes as research has shown that the effects of PA interventions tend to be short-lived (80, 81). Finally, the underlying mechanism through which an older adult's caregiving to his/her spouse increases the probability of MVPA initiation of this older adult is unclear. While fearing for burdening family members for caregiving in the future might motivate a PA change of this older adult, we are unable to test such mechanism directly due to data limitations. Future research using a qualitative method might help identify the underlying mechanism for the positive effect, which would inform effective PA interventions.

Despite its limitations, this study contributes to the literature by using longitudinal data and applying an IV approach with an innovative instrument (i.e., spousal falls). We identified a positive causal effect of providing care to one spouse on the initiation of MVPA of the other spouse among older adults, especially women and non-Hispanic whites. The positive impact might be the result of vicarious experience and learning about caregiving burden. This study has important implications for policymakers and public health and healthcare professionals. Considering the positive effect of spousal caregiving on individuals' PA, providing PA-promoting education using a family-focused approach (82) may be more effective to reach older adults. For example, information about caregiving burden and the harm of a sedentary lifestyle can be shared with the family. Such information perhaps would lead to a change in the caregiver's health behavior, as research has suggested the effectiveness of information with a focus on the salience of events which have happened to family members on individuals' behavioral change (83). Family events can be leveraged as a window of opportunity for targeted interventions (83), and caregivers may be more prone to positive behavioral changes when they are offered risk and educational messages on PA when these events happen (e.g., information offered by doctors of their ill spouses). Our results suggest that this strategy could be effective especially for female and non-Hispanic white older adults as we found positive effects of spousal caregiving on their MVPA initiation. PA-promoting interventions can further benefit these older adults who are prone to the positive behavioral change in PA due to caregiving. For male and non-white older adults, although this strategy might not be as effective, they should still be reminded of caregiving burden and the risk of lack of PA. The efficacy of health promotion messages delivered to these older adults might be strengthened if accompanied by programs to shift the social norms and expectations about caregiving roles.

## Data availability statement

Publicly available datasets were analyzed in this study. This data can be found here: https://hrs.isr.umich.edu/data-products.

## Author contributions

Both authors listed have made an equal, substantial, direct, and intellectual contribution to the work and approved it for publication.
