# Supplementary material for: The positive impact of informal spousal caregiving on the physical activity of older adults
Source: Front Public Health. 2022 Dec 16;10:977846. doi: 10.3389/fpubh.2022.977846 (PMC9800888; doi:10.3389/fpubh.2022.977846)
Supplement: Supplementary file 1 [file Table_1.docx]

**Supplemental materials**

**Table A1. Details of variables used in the analysis**

| **Variables** | **Coding** |
| --- | --- |
| **Dependent variable** |  |
| PA initiation | 1if a respondent reports engaging in moderate-or-vigorous PA at least one to three times a month at the current period, but not in the previous period, and 0 if otherwise |
| **Explanatory variable** |  |
| Informal care for spouse | 1 if a respondent assists their spouse in performing ADLs or IADLs, and 0 if otherwise. |
| **Instrument variable** |  |
| Fall | 1 if a respondent’s spouse (age ≥ 65) reported having fallen down in the previous two years, and 0 if otherwise |
| Non-injurious fall | 1 if a respondent’s spouse (age ≥ 65) reported having fallen down but did not need medical treatment in the previous two years, and 0 if otherwise |
| Injurious fall | 1 if a respondent’s spouse (age ≥ 65) reported having fallen down and needed medical treatment in the previous two years, and 0 if otherwise |
| **Control variable** |  |
| *Biological constraints* |  |
| Medical conditions | Nine dummy variables indicating a doctor’s diagnosis of high blood pressure, diabetes, cancer, lung disease, heart problem, stroke, arthritis, psychiatric problem, and memory-related disease. |
| Self-reported health | Dummy variables indicating poor, fair, very good, and excellent (ref.= good) |
| Difficulties with ADLs | Number of reported difficulties with walking across a room, dressing, bathing/taking a shower, getting in/out of bed, eating, getting in/out of bed, or using the toilet (score 0-7) |
| Difficulties with IADLs | Number of reported difficulties with using a map, using the phone, managing money, taking medications, shopping for groceries, or preparing hot meals (score 0-7) |
| CES-D score | Center for Epidemiologic Studies Depression Scale (CES-D) score for mental health (score 0-8) |
| *Time constraints* |  |
| Employment status | Dummy variables indicating being employed, not working, and retired (ref.= not working) |
| % contribution to income | The percentage contribution of respondents’ earnings to total household income capturing the opportunity cost of their time. |
| Number of children | The number of living adult children |
| *Time and risk preferences* |  |
| Current smoker | 1 if current smoking status, and 0 if otherwise |
| Number of drinks per week | Natural log of the number of alcoholic drinks per week addressing its highly skewed distribution. |
| Weight status | Dummy variables indicating being underweight (below 18.5), normal (18.5-24.9), overweight (25.0-29.9), and obese (30.0 and above) based on Body Mass Index (ref.= normal) |
| *Other control variables* |  |
| Age | Age and age squared |
| year and month of interview | Account for seasonal and regional trends, and potential barriers to outdoor exercise |
| census region | Dummy variables indicating being in Midwest, South, and West (ref.= Northeast), accounting for seasonal and regional trends, and potential barriers to outdoor exercise |
| Household income | Natural log household income. The values are inflation adjusted based on the Consumer Price index. |
| Household net worth | Inverse hyperbolic sine of net worth to handle the skewed distribution with non-positive values (Pence, 2006) |
| Health insurance ownership | 1 if a respondent is covered by federal government health insurance program, insurance from current or previous employers, or other health insurance, and 0 if otherwise |
| Home ownership | 1 if a respondent owns a home, and 0 if otherwise |
| Spouse’s medical condition | Nine indicators of reporting a doctor’s diagnosis of high blood pressure, diabetes, cancer, lung disease, heart problem, stroke, arthritis, psychiatric problem, and memory-related disease |
| **Other variables** |  |
| Self-assessed longevity | Self-reported probability of living another 10 years |
| Self-assessed formal long-term care use | Self-reported probability of entering into a nursing home in the next 5 years |
| Sex | Dummy variable indicating whether a respondent is a male or female |
| Race/ethnicity | Dummy variable indicating whether a respondent is a white or non-white individual |

**Table A2. Test for heterogeneity in the relationship by type of care**

|  | Specification (I) | | Specification (II) | |
| --- | --- | --- | --- | --- |
|  | 1st | 2nd | 1st | 2nd |
|  | Coef.  (S.E.) | Coef.  (S.E.) | Coef.  (S.E.) | Coef.  (S.E.) |
| Spousal fall | 0.3449***  (0.0037) |  | 0.0224***  (0.0043) |  |
| Help performing ADLs |  | 0.3045*  (0.1284) |  |  |
| Help performing IADLs |  |  |  | 0.4684*  (0.2112) |
| Obs. | 26,227 | 26,227 | 26,227 | 26,227 |
| N | 9,173 | 9,173 | 9,173 | 9,173 |
| Cragg-Donald Wald F statistic | 89.01 | | 27.18 | |

*Note*. Individual-fixed effects 2SLS estimators. Covariates are controlled in all specifications. * *p*<.05, ** *p*<.01, *** *p*<.001

**Table A3. Test for random assignments of spousal fall variables to respondents’ characteristics**

|  | (I) Fall | (II) Non-injurious fall | (III) Injurious fall |
| --- | --- | --- | --- |
|  | Coef.  (S.E.) | Coef.  (S.E.) | Coef.  (S.E.) |
| Age | -0.0361*  (0.0153) | -0.0223  (0.0143) | -0.0138  (0.0102) |
| Age squared (/1,000) | 0.1910**  (0.0725) | 0.0807  (0.0677) | 0.1103*  (0.0484) |
| Self-reported health (ref.= good) |  |  |  |
| Poor | 0.0483*  (0.0211) | 0.0261  (0.0197) | 0.0222  (0.0141) |
| Fair | 0.0001  (0.0115) | -0.0011  (0.0107) | 0.0012  (0.0077) |
| Very good | -0.0071  (0.0091) | -0.0019  (0.0085) | -0.0051  (0.0060) |
| Excellent | -0.0062  (0.0154) | -0.0067  (0.0144) | 0.0005  (0.0102) |
| Diagnoses with medical conditions |  |  |  |
| High blood pressure | -0.0058  (0.0152) | 0.0030  (0.0142) | -0.0088  (0.0101) |
| Diabetes | 0.0092  (0.0179) | 0.0035  (0.0167) | 0.0056  (0.0119) |
| Cancer | -0.0234  (0.0188) | -0.0338  (0.0176) | 0.0104  (0.0125) |
| Lung disease | 0.0024  (0.0225) | 0.0097  (0.0210) | -0.0073  (0.0150) |
| Heart problem | 0.0347*  (0.0154) | 0.0295*  (0.0144) | 0.0052  (0.0103) |
| Stroke | -0.0336  (0.0273) | -0.0342  (0.0255) | 0.0006  (0.0182) |
| Arthritis | -0.0342*  (0.0148) | -0.0215  (0.0138) | -0.0127  (0.0098) |
| Psychiatric problem | 0.0259  (0.0232) | 0.0107  (0.0216) | 0.0152  (0.0155) |
| Memory-related disease (MRD) | -0.0215  (0.0252) | -0.0047  (0.0235) | -0.0168  (0.0168) |
| Difficulties with performing ADLs |  |  |  |
| Walking across a room | 0.0594**  (0.0222) | 0.0525*  (0.0207) | 0.0069  (0.0148) |
| Dressing | 0.0168  (0.0167) | -0.0004  (0.0156) | 0.0172  (0.0111) |
| Bathing/taking a shower | -0.0140  (0.0248) | -0.0159  (0.0231) | 0.0019  (0.0165) |
| Eating | 0.0300  (0.0332) | 0.0298  (0.0310) | 0.0002  (0.0221) |
| Getting in/out of bed | 0.0223  (0.0228) | 0.0026  (0.0213) | 0.0198  (0.0152) |
| Using the toilet | 0.0107  (0.0207) | 0.0285  (0.0193) | -0.0178  (0.0138) |
| Difficulties with performing IADLs |  |  |  |
| Using a map | 0.0080  (0.0146) | 0.0170  (0.0136) | -0.0091  (0.0097) |
| Using the phone | -0.0030  (0.0275) | -0.0108  (0.0256) | 0.0078  (0.0183) |
| Managing money | -0.0720**  (0.0251) | -0.0447  (0.0234) | -0.0273  (0.0167) |
| Taking medications | -0.0216  (0.0320) | 0.0331  (0.0298) | -0.0547*  (0.0213) |
| Shopping for groceries | 0.0216  (0.0207) | 0.0147  (0.0193) | 0.0069  (0.0138) |
| Preparing hot meals | -0.0168  (0.0236) | -0.0116  (0.0220) | -0.0052  (0.0157) |
| CES-D score | 0.0000  (0.0028) | -0.0025  (0.0026) | 0.0025  (0.0019) |
| Employment status (ref= unemployed) |  |  |  |
| Employed | 0.0005  (0.0207) | 0.0029  (0.0193) | -0.0024  (0.0138) |
| Retired | 0.0025  (0.0160) | 0.0072  (0.0149) | -0.0047  (0.0106) |
| % contribution to household income | 0.0069  (0.0298) | -0.0049  (0.0278) | 0.0117  (0.0199) |
| Health insurance owners | 0.0000  (0.0181) | 0.0135  (0.0168) | -0.0135  (0.0120) |
| Natural log of income | 0.0076  (0.0055) | 0.0155**  (0.0051) | -0.0080*  (0.0037) |
| Homeownership | 0.0005  (0.0219) | 0.0174  (0.0204) | -0.0169  (0.0146) |
| Inverse hyperbolic sine of net worth | -0.0047  (0.0044) | -0.0045  (0.0041) | -0.0002  (0.0029) |
| Number of children | -0.0109  (0.0084) | -0.0072  (0.0078) | -0.0037  (0.0056) |
| Current smokers | -0.0100  (0.0270) | 0.0150  (0.0251) | -0.0249  (0.0180) |
| Natural log of number of drinks | -0.0005  (0.0072) | 0.0105  (0.0067) | -0.0111*  (0.0048) |
| Weight status (ref.= normal) |  |  |  |
| Under-weight | 0.0363  (0.0411) | 0.0008  (0.0383) | 0.0355  (0.0274) |
| Overweight | -0.0116  (0.0134) | -0.0167  (0.0125) | 0.0051  (0.0090) |
| Obese | -0.0063  (0.0181) | -0.0238  (0.0168) | 0.0175  (0.0120) |
| Obs. | 26,227 | 26,227 | 26,227 |
| N | 9,173 | 9,173 | 9,173 |
| R-squared | 0.0264 | 0.0150 | 0.0116 |

*Note*. Fixed-effect regression estimators. We control for year-, month-, region-, and individual-fixed effects. * *p* < 0.05, ** *p* < 0.01, *** *p* < 0.001
